# Strain-Modulated and Nanorod-Waveguided Fluorescence in Single Zinc Oxide Nanorod-Based Immunodetection

**DOI:** 10.3390/bios14020085

**Published:** 2024-02-03

**Authors:** Marion Ryan C. Sytu, Andrew Stoner, Jong-In Hahm

**Affiliations:** Department of Chemistry, Georgetown University, 37th & O Sts. NW., Washington, DC 20057, USA

**Keywords:** ZnO nanorod, strain, compression, tension, fluorescence, immunodetection, subwavelength waveguiding, protein sensor, biosensor

## Abstract

Mechanical strain has been shown to be a versatile and tunable means to control various properties of nanomaterials. In this work, we investigate how strain applied to individual ZnO nanorods (NRs) can affect the fluorescence signals originated from external sources of bioanalytes, which are subsequently coupled and guided onto the NRs. Specifically, we determine how factors such as the NR length and protein concentration can influence the strain-induced changes in the waveguided fluorescence intensity along the NRs. We employ a protein of tumor necrosis factor-α (TNF-α) and a fluorophore-labeled antibody in a model immunoassay reaction, after which Alexa488-TNF-α immunocomplex is formed on ZnO NRs. We elucidate the relationships between the types as well as amounts of strain on the NRs and the fluorescence intensity originated from the Alexa488-TNF-α immunocomplexes. We show that tensile (compressive) strain applied to the NR leads to an increase (decrease) in the waveguided fluorescence signals. By assessing important optical phenomena such as fluorescence intensification on nanorod ends (*FINE*) and degree of *FINE* (*DoF*), we confirm their linear dependence with both the types and amounts of strain. Furthermore, the strain-induced changes in both *FINE* and *DoF* are found to be independent of protein concentration. We determine that NR length plays a critical role in obtaining high strain-dependence of the measured fluorescence signals. Particularly, we ascertain that longer NRs yield larger changes in both *FINE* and *DoF* in response to the applied strain, relative to shorter ones. In addition, longer NRs permit higher linear correlation between the protein concentration and the waveguided fluorescence intensity. These outcomes provide valuable insight into exploiting strain to enhance the detection of optical signals from bioanalytes, thus enabling their quantifications even at ultra-trace levels. Coupled with the use of individual ZnO NRs demonstrated in our measurements, this work may contribute to the development of a miniaturized, highly sensitive biosensor whose signal transduction is best optimized by the application of strain.

## 1. Introduction

Zinc oxide (ZnO) nanostructures have been widely used in various technological fields due to their remarkable intrinsic properties [1]. In particular, ZnO nanorods (NRs) are increasingly recognized in biosensing [2,3,4,5,6], photocatalysis [7,8], and solar cell applications [9,10] due to their extraordinary optical and electrical properties brought on by their high shape anisotropy and reduced dimensionality [1]. In order to enhance their effectiveness in these applications, the ability to adjust the properties of nanomaterials is of paramount importance. Various approaches such as doping [11,12], alloying [13,14], and changing the morphology [15,16] have been employed to modulate NRs’ properties.

One promising strategy involves the use of mechanical strain, which has been previously demonstrated to be a tunable means to control the material’s properties [17,18]. Unlike the aforementioned approaches, the employment of strain can lead to such property changes without introducing chemical impurities or significantly altering the material’s dimensions. It has been shown that the application of mechanical strain is a highly versatile route that can be precisely and reversibly controlled to modify the electronic structure of the material [19,20]. In addition, nanostructures can sustain large amounts of stress, permitting them to have a wider range of tunable property changes compared to bulk materials [20]. As such, previous works have used mechanical strain to modify the resistance [21], near band emission [17,19,20,22], and Schottky barrier height [23,24] of ZnO NWs. Simulation studies based on the finite element method (FEM) and three-dimensional (3D) finite difference time domain (FDTD) have also reported that the optical properties of ZnO NWs, such as reflectance, transmittance, and absorptance, are dependent on the applied mechanical strain [25,26,27]. These previous efforts have demonstrated that the intrinsic optical properties of ZnO nanostructures can be controlled through the application of strain.

Recent uses of ZnO NRs, however, have been extended to optical signals emitted by adjacent sources that are externally coupled to the NRs. The external sources, such as those from multilayered immunoassay complexes formed after a series of protein-antibody reactions, are separated from the NR surface by more than several tens of nm. In the past, ZnO NR arrays have been demonstrated for the multiplexed detection of cytokine biomarkers, interleukin-8 (IL-8), and tumor necrosis factor α (TNF-α), by conjugating them with two different fluorophores [2]. The optical signals from the fluorophore-conjugated cytokines were coupled and guided along the NR’s optical cavity in the array [1]. Upon probing single ZnO NRs, a unique phenomenon termed as fluorescence intensification on nanorod ends (*FINE*) was observed [1,28,29,30,31]. Further studies were carried out to determine nanomaterial and biomolecular factors that affected *FINE* and the degree of *FINE* (*DoF*). From these efforts, key contributors to both *FINE* and *DoF* were revealed as NR length and width, NR orientation with respect to the substrate, the fluorophore’s emission wavelength, protein concentration, and the light polarization [29,30].

Although strain is known to be an important parameter for modulating a material’s properties, as discussed earlier, the relationship between strain and optical signals in nanomaterials is not well-understood to date. For example, very little is known about the role of strain in affecting the abovementioned biomolecular signals waveguided through ZnO NRs. Another example is found in the application of ZnO NRs in flexible settings for light detection [32,33,34], where strain present in these flexible devices can potentially change their functional outcomes. Hence, understanding the strain-dependent NR responses of light signals becomes highly imperative. We have recently undertaken an initial endeavor in this regard to show that the application of strain can influence the light waveguiding behavior of ZnO NRs [31]. However, the previous investigation was limited to the case of organic fluorophores directly placed on the surface of the NR. Further studies are warranted to determine the exact relationships between the strain applied to individual NRs and the waveguided fluorescence from biomolecules that are located many tens of nm away from the NR surface.

Herein, we elucidate the effects of strain on the ZnO NR-waveguided fluorescence signals that originated from a model immunoassay involving TNF-α. TNF-α is an important protein biomarker implicated in inflammatory and autoimmune diseases such as acute kidney injury, rheumatoid arthritis, and psoriasis [2,35], and, hence, is used as a bioanalyte in our study. We first examine the correlation between the protein concentration and the waveguided fluorescence intensity. We then systematically investigate changes in both *FINE* and *DoF* as a function of varying amounts of both compressive and tensile strain. In addition, we ascertain the role of NR length in affecting the responses of both *FINE* and *DoF* versus strain. Our work provides much needed insights into exploiting strain to regulate optical signals from external sources, even when they are not directly placed on the NR surface. The knowledge gained from this study can be used to maximize the detection capabilities of individual ZnO NRs, which can be particularly valuable in the fluorescence-based immunodetection of biomolecules. Furthermore, the outcomes of this study on strain-dependent, optical responses of individual ZnO NRs may contribute to the development of miniaturized biosensors whose strain-tuned detection signals can be optimized even for flexible sensing applications.

## 2. Materials and Methods

TNF-α standard solutions with concentrations of 100, 10, 1, and 0.1 fg/mL were prepared via a series of dilution from recombinant TNF-α (R&D Systems, Minneapolis, MN, USA) in deionized (DI) water. Unlabeled monoclonal anti-human TNF-α antibody (clone 6401, R&D Systems, Minneapolis, MN, USA) was used as a primary capture antibody in our sandwich immunoassay. Lyophilized bovine serum albumin (BSA) was purchased from VWR Scientific, Inc. (West Chester, PA, USA) and dissolved per the manufacturer’s recommendations. Polyclonal anti-human TNF-α antibody (AB 210-NA, R&D Systems, Minneapolis, MN, USA) was labeled with a fluorophore, Alexa488, using the Monoclonal Antibody Labeling kit (Invitrogen Molecular Probes, Eugene, OR, USA) following the manufacturer’s instructions.

ZnO NRs were grown in a home-built, horizontal tube furnace via the chemical vapor deposition (CVD) method. Briefly, a colloidal solution of 20 nm Au NPs (Ted Pella, Inc., Redding, CA, USA) was deposited on a Si plate (Silicon Quest International Inc., San Jose, CA, USA) as the growth catalyst. The source material for ZnO NR growth was a 1:2 by weight mixture of 99.999% ZnO and 99% graphite powders (Alfa Aesar Inc., Tewksbury, MA, USA). A quartz boat containing the source material was placed at the center of the tube furnace. The Si wafer with pre-deposited Au catalysts was placed 15 cm downstream from the source boat. The horizontal tube furnace was heated to 950 °C for 15 min to 1 h at a rate of 15 °C/min under a constant Ar flow at a rate of 100 standard cubic centimeters per minute. Pristine ZnO NRs were imaged using a FEI/Philips XL 20 scanning electron microscope (SEM) operated at 20 keV for morphological characterization.

The as-synthesized ZnO NRs were sonicated in ethanol and drop-casted on a clean Si wafer. A 15 µL aliquot of 1 µg/mL solution of TNF-α unlabeled primary antibody was deposited on the ZnO NR platform for 15 min and then thoroughly rinsed with DI water. The unreacted sites on the ZnO NRs were blocked with 15 µL of 1% (*w*/*v*) BSA solution for 20 min, after which the unbound BSA was washed off with DI water. Subsequently, a 15 µL aliquot of TNF-α standard solution with varying concentrations of 100, 10, 1, and 0.1 fg/mL was added on the ZnO NR platform. After a 15 min incubation period, the platform was rinsed with DI water. Lastly, the ZnO NR platform was incubated with 15 µL of 1.5 µg/mL solution of Alexa488-labeled TNF-α antibody for 30 min and rinsed with DI water. All reactions were carried out in a humidity-controlled chamber, protected from light at room temperature. The sandwich immunoassay resulted in the formation of Alexa488-TNF-α immunocomplex, composed of unlabeled TNF-α capture antibodies/TNF-α/Alexa488-labeled TNF-α secondary antibodies. The resulting Alexa488-TNF-α immunocomplex/ZnO NRs on the Si plate was dried gently with a stream of N_2_ gas.

The Alexa488-TNF-α immunocomplex/ZnO NRs on the Si plate was placed face up on a petri dish. A polydimethylsiloxane (PDMS) elastomer mixture (Dow Corning, Midland, MI, USA) was prepared and added to the petri dish. The PDMS elastomer mixture was then cured at 80 °C for 24 h. This process led to the transfer of Alexa488-TNF-α immunocomplex/ZnO NRs to the cured PDMS. ZnO-PDMS constructs prepared by a method similar to that described above have been successfully used to fabricate stretchable and flexible sensors in the past [36,37,38]. The elastomeric piece of PDMS containing Alexa488-TNF-α immunocomplex/ZnO NRs was carefully peeled off from the Si plate. The resulting Alexa488-TNF-α immunocomplex/ZnO NRs/PDMS was mounted on a microvice (S.T. Japan USA LLC, Ft. Myers, FL, USA), which was employed for applying uniaxial compressive and tensile stress. All measurements were performed under ambient conditions.

Fluorescence measurements were carried out using a Zeiss Axio Imager A2M microscope (Carl Zeiss, Inc., Jena, Germany) with an AxioCAM HRm digital camera (Carl Zeiss, Inc., Jena, Germany). The source of fluorescence excitation was a 120 W Hg lamp (X-Cite 120Q, Carl Zeiss, Inc., Jena, Germany). The fluorescence emission from the Alexa488-TNF-α immunocomplex coupled to ZnO NRs was acquired using an EC Epiplan-NEOFLUAR objective lens (50×, numerical aperture of 0.8) using a spectroscopic filter cube (λ_excitation_ = 450–490 nm, λ_emission_ = 510–540 nm) at a 5 s exposure. The use of high optical-quality ZnO NRs grown via CVD prevented any spectral overlap with the absorption and emission characteristics of the fluorophores, thus ensuring accurate measurements of the fluorescence intensities emitted by the Alexa488-TNF-α immunocomplex. Fluorescence intensities were acquired and analyzed using software applications such as Zeiss AxioVision, Image J, and Origin 9.

## 3. Results and Discussion

**Figure 1:**
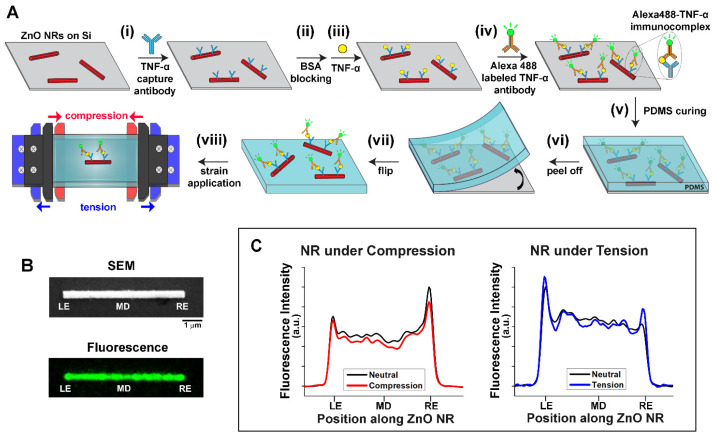
(**A**) The scheme illustrates the overall fabrication process of the TNF-α sandwich immunoassay based on ZnO NRs. The NR immunoassay platform was integrated onto a PDMS elastomer for the application of uniaxial tensile (blue) and compressive (red) strain with a microvice during fluorescence measurements. (**B**) Representative SEM (**top**) and fluorescence (**bottom**) images of ZnO NRs are shown. On both images, the left-end, middle, and right-end positions of the NR are denoted as LE, MD, and RE, respectively. In the fluorescence image, the emission from the Alexa488-TNF-α immunocomplex is pseudo-colored green. (**C**) The two plots show changes in waveguided fluorescence intensity as a function of position on a ZnO NR when uniaxial strain was applied to the NR. The left plot displays a decrease in the overall fluorescence intensity with compressive (red) strain, compared to the neutral state (black). The right plot presents the case for tensile (blue) strain where an increase in the fluorescence intensity at both NR ends was observed relative to the neutral state (black).

**Fabrication of Alexa488-TNF-α immunocomplex/ZnO NRs/PDMS Constructs.** Figure 1A displays the overall process of fabricating a TNF-α immunoassay construct based on ZnO NRs and its subsequent transfer to a PDMS substrate, i.e., Alexa488-TNF-α immunocomplex/ZnO NRs/PDMS. ZnO NRs in ethanol were drop-casted on a Si wafer, onto which unlabeled TNF-α capture antibodies were immobilized, as presented in step (i). The capture antibodies were coupled to the surface of the ZnO NRs via physical adsorption. This method has been previously utilized to successfully link different antibodies to ZnO NRs for immunodetection [39,40]. Any unreacted areas of both ZnO NRs and Si were blocked by BSA, and the pre-made TNF-α standards with different concentrations were introduced afterwards in steps (ii) and (iii), respectively. Subsequently, Alexa488-labeled TNF-α secondary antibodies were deposited for a sandwich type of immunoassay reaction, as shown in step (iv). The series of bioreactions resulted in the formation of the Alexa488-TNF-α immunocomplex. Following the TNF-α immunoassay reaction, a PDMS mixture was cured on top of the sandwiched protein assembly on the Si wafer in step (v). The PDMS construct, along with ZnO NR immunoassay platform, was peeled off from the Si to expose the sandwich construct on top of the elastomer piece for our optical detection in steps (vi) and (vii). The use of a PDMS elastomer enables the applications of uniaxial compressive (red) and tensile (blue) strain using a microvice as depicted in step (viii).

**Representative Data from Alexa488-TNF-α immunocomplex/ZnO NRs Under Strain.** Figure 1B shows a representative SEM image of pristine ZnO NRs in the top panel. The bottom panel of Figure 1B displays a representative fluorescence image of ZnO NRs upon performing the TNF-α immunoassay reactions depicted in Figure 1A. The emission from the Alexa488-TNF-α immunocomplexes, coupled into the NR cavity and guided along the length of the NR, is clearly visible along the length of the NR in the fluorescence panel of Figure 1B. The effects of uniaxial strain on the waveguided fluorescence intensity on ZnO NRs were then elucidated. Figure 1C displays representative plots of fluorescence intensity measured from individual ZnO NRs under uniaxial strain. The fluorescence intensity changes are displayed as a function of different positions along given NRs when the NRs were subject to compressive and tensile strain in the left and right panel, respectively, relative to the strain-free state. When NRs were under compressive strain (red), the coupled emission from the Alexa488-TNF-α immunocomplex decreased along all the positions of the NR, relative to the unstrained state (black). In contrast, the application of tensile strain (blue) caused the coupled fluorescence along the NR to increase at both NR ends, when compared to its strain-free state (black). All fluorescence intensity values reported in this paper were subtracted by those blank signals measured without TNF-α. This trend of changes in fluorescence intensity with strain was repeatedly observed from approximately 300 ZnO NRs across different PDMS constructs.

**Figure 2:**
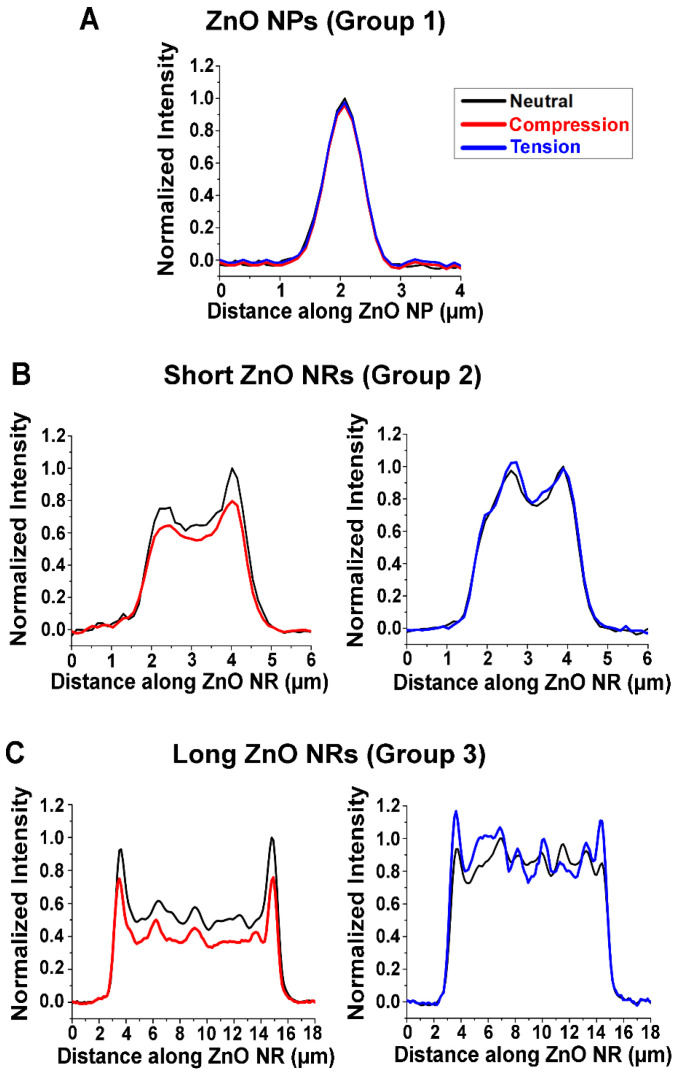
Representative plots of normalized fluorescence intensity with respect to position along individual (**A**) ZnO NPs, (**B**) short ZnO NRs, and (**C**) long ZnO NRs under neutral (black), compressive (red), and tensile (blue) strain. (**A**) ZnO NPs (Group 1) show no changes in the waveguided fluorescence intensity under compressive and tensile strain compared to the strain-free case. (**B**) Short ZnO NRs (Group 2) present a decrease in fluorescence intensity with compressive strain, while a slight increase in fluorescence intensity is observed with tensile strain. (**C**) Long ZnO NRs (Group 3) display similar trends as Group 2, but they exhibit greater *FINE* that is clearly observed at the two NR ends compared to the shorter NRs in Group 2. The fluorescence intensity of the NRs in Group 3 decreased with compressive strain. For the case of tension, the Group 3 NRs exhibited pronounced signal increases at the two NR ends.

**Influence of NR Length on Waveguided Fluorescence under Strain.** When varying strain, three distinct responses were observed for the waveguided fluorescence intensity of the ZnO nanomaterials. We categorized these into three different groups of Groups 1, 2, and 3. Figure 2 shows the representative plots of normalized fluorescence intensity with respect to the position along individual ZnO nanomaterials for all three groups, when they were under strain-free (black), compression (red), and tension (blue). When compressive and tensile strain were applied to those in Group 1, the fluorescence intensity remained unchanged relative to the strain-free state. This behavior, shown in Figure 2A, was observed in ZnO nanomaterials that have very short lengths of <1 µm, i.e., ZnO nanoparticles (NPs). For Group 2, the waveguided fluorescence intensity decreased with compressive strain, while a slight increase in intensity was detected with tensile strain. ZnO NRs of ~1–3 µm in length exhibited this behavior, which is shown in Figure 2B. The ZnO NPs and the short ZnO NRs employed in our study have similar structures to those reported in our previous study [41]. NRs belonging to Group 3 presented the same general trend as those in Group 2, where compressive strain caused a decrease in the waveguided fluorescence intensity from the Alexa488-TNF-α immunocomplex. The Group 3 NRs undergoing tension, on the other hand, clearly exhibited noticeable increases in fluorescence signals at the two NR ends. We observed this behavior in ZnO NRs that are longer, ~5–20 µm in length, which is presented in Figure 2C. A representative SEM image of the long ZnO NRs is displayed in Figure 1B. Due to the instrumental limitation of the microvice apparatus used for the application of strain, NRs of ~20 µm at maximum could be examined in our study, beyond which fell outside the mechanical operation range of the microvice for the effective production of strained NRs. The extended length of the NRs led to more effective waveguiding of fluorescence, thus producing the pronounced signal changes at the two NR ends under tension. These results are repeatedly observed from a total of 75 ZnO NPs, 60 short ZnO NRs, and 230 long ZnO NRs that are categorized as Group 1, Group 2, and Group 3, respectively.

Compared to the short NRs in Group 2, the long NRs in Group 3 exhibited a more distinct *FINE* effect. Longer NRs have been previously reported to have better propagation of the guided light along the NR cavity, resulting in higher *DoF* values [29]. This was also supported by a FDTD simulation of a single emitter on a ZnO NR [29]. This study revealed that a longer NR has a greater and highly directional emission along the long axis of the NR, enhancing the *FINE* effect. Our results are demonstrated in Figure 2B,C, where longer NRs are associated with higher *DoF*s relative to shorter ones, once again emphasizing the role that the NR length plays on the waveguided fluorescence signal from bioanalytes coupled to ZnO NRs. As such, the lack of strain-dependent response in fluorescence intensity observed from the NPs in Figure 2A is due to their very short lengths and high shape isotropy, making them less effective for waveguiding the fluorescence signal.

**Figure 3:**
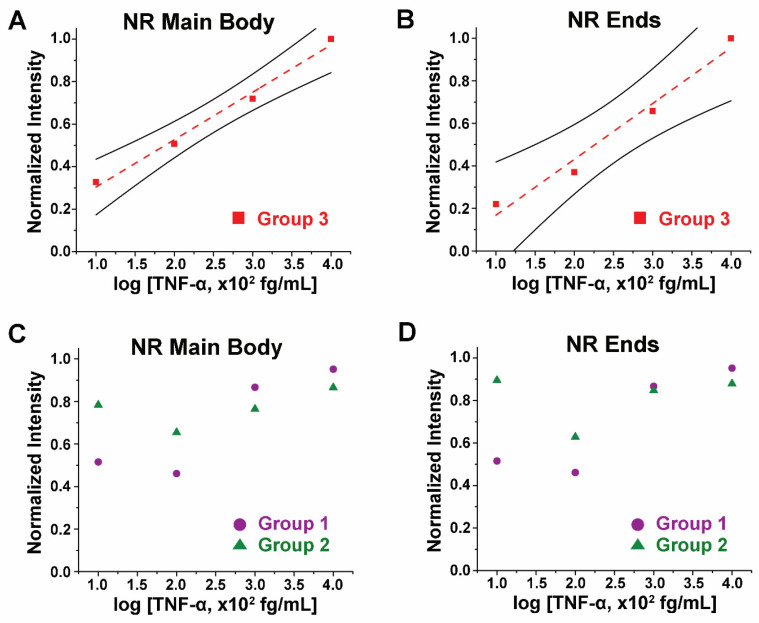
The plots display normalized fluorescence intensity with respect to TNF-α concentration on (**A**) NR main body and (**B**) NR ends from the long NRs in Group 3 (red square). The Group 3 NRs show an increasing trend in fluorescence intensity with increasing TNF-α concentration on both (**A**) NR main body and (**B**) NR ends. The red dashed lines are linear fits for the data points, while the solid black lines above and below the data points represent the 95% confidence intervals. For comparison, the NPs in Group 1 (purple circle) and short NRs in Group 2 (green triangle) were similarly evaluated. Plots showing normalized fluorescence intensity as a function of TNF-α concentration on (**C**) NR main body and (**D**) NR ends are then presented. Group 1 NPs and Group 2 NRs do not exhibit any significant correlations between the fluorescence intensity and TNF-α concentration either from (**C**) NR main body or (**D**) NR ends.

**TNF-α Concentration and Waveguided Fluorescence under Strain-Free States.** The biomolecular concentration dependence of the waveguided fluorescence intensity was first examined without strain. Different TNF-α concentrations of 100, 10, 1, and 0.1 fg/mL were incorporated into our sandwich immunoassay reactions shown in Figure 1A. Fluorescence intensities from the Alexa488-TNF-α immunocomplex coupled to long NRs in Group 3 were obtained and analyzed. NPs in Group 1 and short NRs in Group 2 were also prepared with different TNF-α concentrations, where the obtained fluorescence intensities from the sandwich immunoassay were compared to those obtained from the long NRs in Group 3. Figure 3A,B show the plots of the TNF-α concentrations versus normalized fluorescence intensities on the NR main body and ends, respectively, from the long NRs in Group 3 (red square). From both plots, the normalized fluorescence intensity increased with higher TNF-α concentrations. The regressions (red dashed lines) display linear trends of the fluorescence intensities with TNF-α levels, both on the NR main body and NR ends.

Figure 3C,D combinedly present the plots of TNF-α concentrations versus normalized fluorescence intensities on the NR main body and ends, respectively, for the case of the NPs in Group 1 (purple circle) and the short NRs in Group 2 (green triangle). For the isotropic NPs in Group 1, average intensities observed from the whole NP were used both for the main body as well as the ends for given NPs. From both plots, no apparent trend between the fluorescence intensity and TNF-α levels was observed from the NPs in Group 1 and the short NRs in Group 2. Our data indicate that the linear trend between the TNF-α concentration and the waveguided fluorescence signal is more evident for the longer NRs. The superior waveguiding capability of the longer NRs, compared to the shorter ones, enabled facile detection of very small changes in protein concentration, down to the 0.1 fg/mL level. Hence, the length of the ZnO NR plays a critical role in the high sensitivity needed for the accurate detection of biomolecules.

**Influence of TNF-α Concentration on Strain-Induced Changes in *FINE*.** We further examined the degrees to which *FINE* of the Group 3 NRs are affected by uniaxial strain at different protein concentrations. To systematically analyze this, we quantitatively measured changes in *FINE* with and without applied strain to the NRs. The % *FINE* and % strain in NR length were first calculated using Equations (1) and (2), respectively. *I_ends_* is the average fluorescence intensity at the NR ends. The subscript ‘strain’ refers to either compression or tension, whereas the subscript ‘neutral’ indicates a strain-free state. The values of % strain were calculated based on the changes in the NR length, before and after strain was applied. *L_strain_* is the length of the NR under strain and *L_neutral_* is the original length of the NR before strain. Negative values of % strain pertain to compression, whereas positive values correspond to tension.

(1) $$\%\;\mathrm{FINE}=\frac{I_{\mathrm{ends},\mathrm{strain}}-I_{\mathrm{ends},\mathrm{neutral}}}{I_{\mathrm{ends},\mathrm{neutral}}}\times 100\%$$

(2) $$\%\;\mathrm{strain}\;\mathrm{in}\;\mathrm{NR}\;\mathrm{length}=\frac{L_{\mathrm{strain}}-L_{\mathrm{neutral}}}{L_{\mathrm{neutral}}}\times 100\%$$

**Figure 4:**
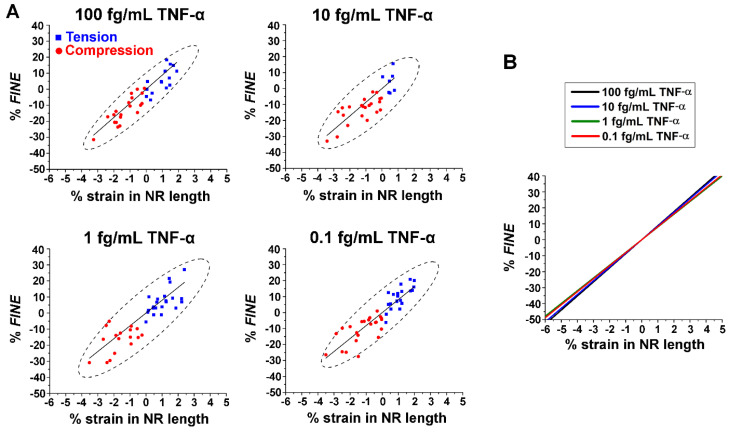
(**A**) The plots display % *FINE* versus % strain in NR length for TNF-α concentrations of 100, 10, 1, and 0.1 fg/mL. Squared data points in blue correspond to the NRs undergoing tension, while circled data points in red refer to the NRs undergoing compression. The solid black lines represent the linear fits for the data points, while the black dashes show the 95% confidence ellipses. Positive values on the x axis of % strain in NR length denote tension, while negative values indicate compression. (**B**) The plot combinedly shows regressions from % *FINE* versus % strain for all TNF-α concentrations in (**A**). The overlaid regression lines exhibit linear relationships of similar slopes between % *FINE* and % strain in NR length.

The calculated values of % *FINE* were subsequently plotted against % strain in NR length for various TNF-α concentrations. The results are presented in a series of plots in Figure 4A. Relative to the neutral state at 0% strain, compressions on the NRs (red circle) decreased the waveguided fluorescence intensity at the NR ends, which led to negative values of % *FINE*. Higher levels of compressive strain caused further decreases in fluorescence intensity at the NR ends. In contrast, higher % tensile strains (blue square) resulted in greater intensification of the fluorescence intensity at the NR ends, producing more positive values of % *FINE*. The regressions (solid black lines) suggest a linear relationship between the change in *FINE* and the types as well as amounts of strain applied to the NR. The plot in Figure 4B displays overlaid linear fits for all TNF-α concentrations from Figure 4A. The intensity values of the fluorescence observed at the NR ends are dependent on protein concentrations, as revealed in Figure 3B. Intriguingly, all regression lines in Figure 4B lie close together with similar slopes. This further indicates that similar levels of linear responses can be expected between strain and the corresponding change in *FINE*, regardless of the protein concentration. It is also clear from Figure 4B that the degree to which compressive/tensile strain can decrease/increase the fluorescence intensity from the strain-free state is just as effective for the lowest TNF-α concentration as for the highest concentration we tested.

**Influence of TNF-α Concentration on Strain-Induced Changes in *DoF*.** Similarly, we quantitatively measured the changes in *DoF* with and without applied strain to individual NRs in Group 3. *DoF* is a measure of the degree of fluorescence intensification observed on the NR ends relative to that on the NR main body. As shown in Equation (3), the *DoF* is calculated as the difference in average fluorescence intensities measured at the ends (*I_avg,end_*) and on the main body (*I_avg,body_*), divided by the average fluorescence intensity on the main body (*I_avg,body_*). The *DoF* values at the neutral state (*DoF_neutral_*) and at the strained state (*DoF_strain_*) of a given NR were then used to determine % *DoF* with Equation (4).

(3) $$\mathrm{DoF}=\;\frac{I_{\mathrm{avg},\mathrm{end}}-I_{\mathrm{avg},\mathrm{body}\;}}{I_{\mathrm{avg},\mathrm{body}\;}}$$

(4) $$\%\;\mathrm{DoF}=\;\frac{{\mathrm{DoF}}_{\mathrm{strain}\;}-\;{\mathrm{DoF}}_{\mathrm{neutral}\;}}{{\mathrm{DoF}}_{\mathrm{neutral}\;}}\times 100\%$$

**Figure 5:**
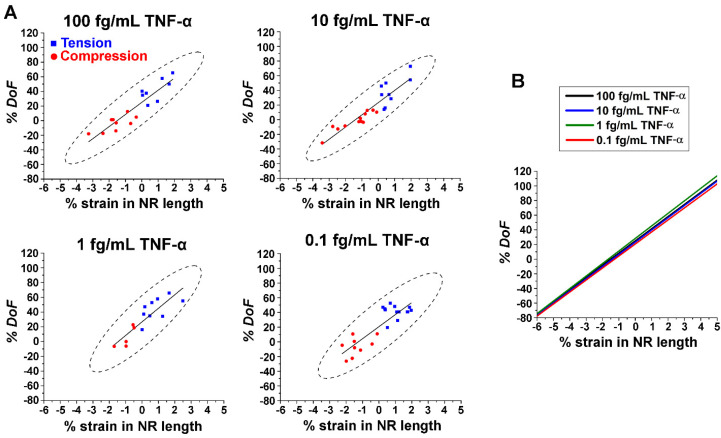
(**A**) The plots display % *DoF* versus % strain in NR length for TNF-α concentrations of 100, 10, 1, and 0.1 fg/mL. Squared data points in blue correspond to the NRs undergoing tension, while circled data points in red refer to the NRs undergoing compression. The solid black lines represent the linear fits for the data points, while the black dashes show the 95% confidence ellipses. Positive values on the x axis of % strain in NR length denote tension, while negative values indicate compression. (**B**) The plot combinedly shows regressions from % *DoF* versus % strain for all TNF-α concentrations in (**A**). The overlaid regression lines exhibit linear relationships with similar slopes between % *DoF* and % strain in NR length.

The calculated values of % *DoF* were subsequently plotted as a function of % strain in NR length for different TNF-α concentrations. These results are displayed in a series of plots in Figure 5A. Between the two types of strain employed, greater % *DoF* was observed for the NRs undergoing tension (blue square) relative to those under compression (red circle). Regression lines inserted through the data points in Figure 5A also show that the changes in *DoF* are linear with the strain applied to the NRs. The linear fits from Figure 5A are combined from all TNF-α concentrations and displayed as a single plot in Figure 5B. It was revealed that the slopes of the regression lines for the % *DoF* are similar among the different protein concentrations. Regardless of the type of strain applied to the NRs, the absolute values of % *DoF* increased with the magnitude of strain. However, the rate of change in the measured fluorescence intensity is strain-dependent, yet protein concentration-independent. This behavior was observed not only for changes in *FINE* (Figure 4B) but also for those in *DoF* (Figure 5B). We demonstrated in Figure 4 that a small amount of tensile strain, for example, that of ~1.7% applied to the ZnO NR, enabled an increase as high as ~20% in *FINE* from the Alexa488-TNF-α immunocomplex formed from 0.1 fg/mL TNF-α. These results of both *FINE* and *DoF* versus strain collectively confirmed that strain can be a useful tool to expand the NRs’ biomolecular detection capabilities, even for a very low protein concentration down to the range of 0.1 fg/mL.

**Figure 6:**
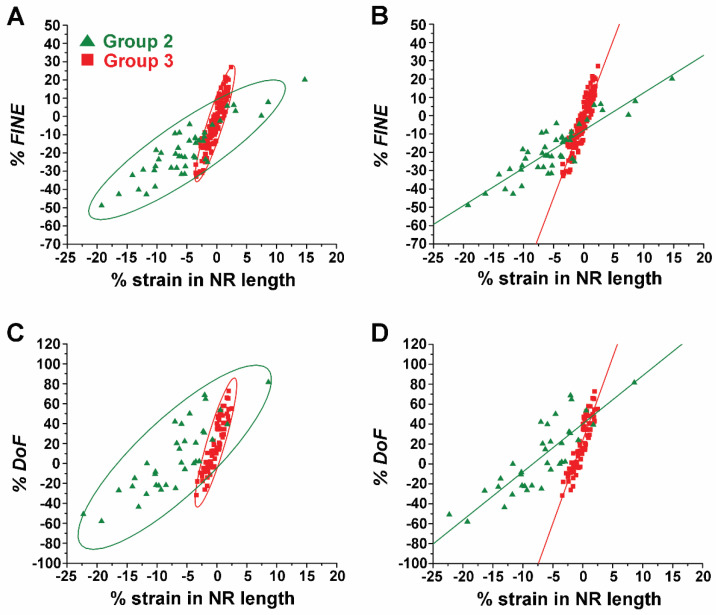
The effect of the NR length was correlated to the strain-induced changes in both *FINE* and *DoF*. (**A**,**B**) The plots display % *FINE* as a function of % strain in NR length for the short NRs in Group 2 (green triangle) and the long NRs in Group 3 (red square). (**C**,**D**) % *DoF* values are plotted as a function of % strain in NR length for the Group 2 NRs (green triangle) and the Group 3 NRs (red square). The red and green circles in (**A**,**C**) belong to 95% confidence ellipses of the Group 2 and Group 3 NRs, respectively. The solid lines in (**B**,**D**) are the respective linear fits through the data points.

**Effect of NR length on Strain-Induced Changes in both *FINE* and *DoF*.** The effects of the NR length on the changes in both *FINE* and *DoF* were further substantiated while individual ZnO NRs were subject to varying types and levels of strain. To systematically analyze the effects of strain, the calculated values of both % *FINE* and % *DoF* of the short NRs in Group 2 and long NRs in Group 3 were compared. We combined all TNF-α concentration data per given NR group in analyzing % *FINE* as well as % *DoF*. This is because, for both NR groups, the strain-induced changes in both *FINE* and *DoF* were independent of the protein concentration. Figure 6A,B show plots of the values of % *FINE* against % strain in NR length for Group 2 (green triangle) and Group 3 (red square). For both groups, a linear dependence between the change in *FINE* and the amount of strain applied is evident from the NRs regardless of the application of tensile or compressive strain. However, the strain-dependent responses from each group differed. This difference can be clearly seen in Figure 6B where the regression line (red) through the data points for the Group 3 NRs exhibits a much steeper slope when compared to that (green) for the Group 2 NRs. At given values of strain, Group 3 NRs showed greater responses in *FINE*, relative to Group 2 NRs. For example, when ~5% tensile strain was applied, the increase in *FINE* for the NRs in Group 3 reached as high as ~45%, whereas only ~3% was observed for those in Group 2. Similar behaviors were also observed under compression. When ~5% compressive strain was applied, a ~45% decrease in *FINE* was measured for the Group 3 NRs, relative to a decrease of only ~18% in *FINE* for the Group 2 NRs.

The plots in Figure 6C,D display % *DoF* as a function of % strain in NR length for the NRs in Groups 2 and 3. The plots for both groups revealed a linear dependence between % *DoF* and % strain. Similar to the behaviors in *FINE* observed between the two groups of NRs, the Group 3 NRs showed greater changes in *DoF* than the Group 2 NRs in response to the applied strain. This tendency in the strain-dependent response of *DoF* can be clearly seen by comparing the slopes of the linear fits for each group, as presented in Figure 6D. Our results corroborate that small amounts of strain can produce substantially higher changes in both *FINE* (Figure 6B) and *DoF* (Figure 6D) for longer NRs.

ZnO NRs were reported to be excellent, low-loss subwavelength waveguides due to their high refractive index (n) throughout the visible spectrum (n > 2) and large bandgap at room temperature [42]. Unlike other forms of ZnO nanostructures, the high shape anisotropy of the NRs is advantageous as optical waveguides for guiding, concentrating, and amplifying light. Individual NR waveguides can be especially beneficial for those applications requiring precise spatial control over light delivery to or collection from highly localized areas. These properties of ZnO NRs have been exploited in numerous optoelectronic and optical devices [1]. A ZnO slab waveguide fabricated on a Si platform was reported to have a relatively low propagation loss (as low as 0.1 dB/cm at λ = 633 nm) compared to other waveguides [43]. Although little is known to date of the full performance characteristics of optical waveguides based on individual NRs, a single NR waveguide made from SnO_2_, which is a transparent conducting oxide material related to ZnO, showed a propagation loss of ~10 dB/cm at λ = 400–550 nm on a Si support [44].

Our results clearly demonstrate a linear relationship between the increase/decrease in the waveguided fluorescence signal and the tension/compression exerted on the individual ZnO NRs. Although further studies are required to understand the exact mechanism of the observed relationship between the strain and optical responses, previous experimental and simulation studies related to strain may provide valuable insights into our experimental observations [19,20,21,22,25,26,27,45]. A study on the photo elastic effect that used a combination of FEM and 3D FDTD modeling simulations reported that the application of mechanical strain can cause a change in refractive index of ZnO nanowires (NWs). This, in turn, results in a phase shift of the transmitted light, optical path of light, and fraction of incident light [26,27]. Previous studies have also reported that the intrinsic bandgap emission of ZnO NWs exhibits a blue (red) shift when the NWs are under compression (tension) [19,20,21,22,45]. These strain-induced changes in bandgaps may be explained by potential changes in the material’s refractive index, where the bandgap of a semiconductor such as ZnO has an inverse relationship with its refractive index [46,47,48]. These previous findings relating strain to changes in refractive index may explain the observed changes in both *FINE* and *DoF* under compressive/tensile strain in this work. The application of compressive (tensile) strain may have decreased (increased) the ZnO NR’s refractive index, which consequently led to a decrease (increase) in the NR’s ability to couple and guide light to its ends by total internal reflection [1].

Our research efforts demonstrate that strain can be used to effectively tune the capability of ZnO NRs as optical waveguides, thus serving as a valuable tool to control both *FINE* and *DoF* on individual ZnO NRs for enhanced biodetection. The application of tensile strain along the ZnO NR can lead to the enhancement of the optical signals waveguided along the NR from a nearby biomolecule, yielding an increased signal that can be easily detected. When considering the dimensions of the ZnO NRs in optimizing their use as a biodetection platform, longer NRs offer greater advantages. Due to the better waveguiding capabilities of longer NRs [29], the waveguided fluorescence intensities on longer NRs are revealed to be more responsive to the changes in protein concentration as well as the application of strain. Even without applied strain, longer NRs greater than 5 µm displayed a linear dependence between biomolecular concentration and waveguided fluorescence intensity. In contrast, shorter NRs and NPs were not as effective to linearly translate changes in biomolecular concentration into those in fluorescence intensity. In addition, longer NRs exhibited more pronounced signal enhancement in response to the applied tensile strain, relative to shorter ones. This signal amplification brought by tensile strain can be just as effectively harnessed for the highest as well as for the lowest protein concentrations we tested. Therefore, the use of longer ZnO NRs along with the application of tensile strain can lead to a much stronger fluorescence signal that is linearly dependent on protein concentration. Our work also demonstrates the critical impact that NR length can play in maximizing the strain-induced changes in the waveguided fluorescence on ZnO NRs. The knowledge gained in this work may be valuable for promoting a novel, strain-modulated application for individual ZnO NRs as an alternative to conventional biodetection platforms by enabling high sensitivity and signal linearity, along with platform miniaturization.

## 4. Conclusions

In summary, we revealed the effect of uniaxial strain on the fluorescence signals that were emitted and guided from the Alexa488-TNF-α immunocomplex along the individual ZnO NRs. Specifically, we assessed the influence of different experimental factors such as NR length, protein concentration, and varying types as well as amounts of strain on the waveguided optical signals. We confirmed that the waveguided fluorescence intensity at the NR ends decreased/increased when the ZnO NRs were under compression/tension. Compared to shorter NRs, longer (>5 µm) NRs showed a more distinct *FINE* due to better waveguiding properties owing to their higher shape anisotropy. In contrast, no strain-induced changes in waveguided fluorescence intensity were observed from ZnO NPs (<1 µm). By employing ZnO nanomaterials with different lengths, we were also able to establish the critical impact of NR length in providing the much-needed sensitivity for accurate protein detection. Regardless of the application of strain, the fluorescence intensity measured on longer NRs exhibited a linear dependence on TNF-α concentration. When strain was applied to individual ZnO NRs, the changes in both *FINE* and *DoF* were found to be linearly dependent on the types and amounts of strain for all protein concentrations tested. Moreover, we ascertained that longer NRs showed more significant changes in both *FINE* and *DoF* in response to the applied strain, relative to shorter ones. Combinedly, the employment of longer NRs under tensile strain can permit protein detection even down to the 0.1 fg/mL range through intensifying the waveguided optical signals to the NR ends. Hence, the NR length and the amount of strain applied to the NR can be synergistically optimized for a highly sensitive detection of biomolecules at ultra-trace levels. Furthermore, strain can be used to modulate waveguided fluorescence signals originated from biomolecules by affecting both *FINE* and *DoF*, regardless of their placement directly on or away from the NR surface. We anticipate that our efforts may motivate the development of miniaturized, individual ZnO NR-based sensors that exploit strain as an effective means to attain a high detection sensitivity as well as a wide linear range in quantifying bioanalytes.

## Data Availability

The data that support the findings of this study are available from the.corresponding author upon reasonable request.
